# Molecular Divergence Between Two Geographically Overlapping Cryptic Crickets: *Loxoblemmus campestris* and *L. sylvestris*

**DOI:** 10.1002/ece3.74457

**Published:** 2026-10-21

**Authors:** Kazuhiro Satomura, Masakazu Saito, Atsushi Ogura

**Affiliations:** ^1^ Department of Bioscience Nagahama Institute of Bioscience and Technology Nagahama Shiga Japan

**Keywords:** cryptic species, genic divergence, molecular evolution, phylogenomics, reproductive isolation, speciation

## Abstract

Understanding the minimal genetic changes underlying the earliest stages of speciation remains a central challenge in evolutionary biology. However, most model systems used to study early speciation involve pronounced ecological divergence, making it difficult to isolate the initial genetic changes associated with speciation. Here, we examined genetic differences between the cryptic cricket species *Loxoblemmus campestris* and *L. sylvestris* , which inhabit the same geographic region without pronounced ecological or morphological divergence. Despite their morphological similarity, the two species exhibited small but consistent molecular phylogenetic divergence in both mitochondrial and nuclear genomes. To investigate genetic divergence between these species, we conducted population genomic and comparative genomic analyses. We identified amino acid substitutions that were common within each species across orthologous genes and evaluated their evolutionary probabilities (*EP*s) based on sequence conservation across Orthoptera. Although low *EP* amino acid substitutions occurred in multiple genes, three genes—*Raf*, *Slit*, and *Sur‐8*—contained multiple such substitutions, and all three also showed elevated *F*_*ST*_ values. These genes are functionally connected within the Ras/MAPK signaling pathway. These findings suggest that genetic divergence between *L. campestris* and *L. sylvestris* may involve changes concentrated in a limited number of functionally connected genes despite relatively few amino acid substitutions and limited phenotypic differentiation. Cryptic *Loxoblemmus* species may therefore provide useful systems for investigating early genomic divergence associated with speciation in the absence of pronounced phenotypic divergence.

## Graphical abstract

We investigated whether genomic divergence between two geographically overlapping, morphologically cryptic crickets, *Loxoblemmus campestri* *s* and *L. sylvestris* , is broadly distributed or concentrated in particular genes and functions. Mitochondrial and nuclear data consistently supported divergence between the two lineages, while relatively few shared amino acid substitutions were detected; notably, evolutionarily rare substitutions were concentrated in *Raf*, *Slit*, and *Sur‐8*, which also showed elevated *F_ST_* values and were highlighted by enrichment analyses. These results suggest that limited sequence divergence between the two lineages may be non‐randomly concentrated in a small set of functionally connected genes, with Ras/MAPK‐associated genes representing candidates for further investigation.

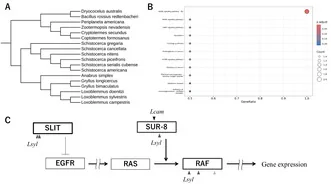

## 1 Introduction

Speciation, the evolution of reproductive isolation between populations, underlies the generation of biological diversity. During speciation, barriers that suppress hybridization between two populations are necessary until complete reproductive isolation is established, and geographical barriers have traditionally been considered crucial (Mayr 1963). However, recent comparative genomic analyses have revealed cases of divergence progressing at both the genetic and phenotypic levels, accompanied by gene flow, even within environments without clear geographic isolation (Schliewen et al. 1994; Barluenga et al. 2006; Savolainen et al. 2006). In such cases, there are ecological and allochronic isolation barriers within local environments, and strong disruptive selection on adaptive traits in different niches is thought to be necessary for divergence in the presence of gene flow (Gavrilets 2004; Feder et al. 2012). In addition to systems where phenotypic divergence is evident, species that are genetically differentiated but exhibit few observable phenotypic differences, so‐called cryptic species, are increasingly recognized (Cheng et al. 2025; Hending 2025; Rani et al. 2025). In particular, it remains unclear how reproductive isolation and genomic divergence can arise and be maintained among cryptic species that coexist within the same habitats.

Insects have been extensively studied as representative models of speciation involving gene flow (Mullen and Shaw 2014). For example, the apple maggot, *Rhagoletis pomonella* , is an insect that is undergoing speciation by shifting its oviposition substrate from wild hawthorn to cultivated apple. The timing of its emergence diverged in order to adapt to the different fruiting periods of these plants (Feder et al. 1994; Filchak et al. 2000). Comparative genomic analysis was expected to reveal the key genomic islands of divergence involved in the early stages of apple maggot speciation. However, it actually suggested that natural selection and divergence occurred across the numerous loci throughout the entire genome (Michel et al. 2010). Stick insects of the genus *Timema* have highly cryptic appearances to evade predators. It is thought that disruptive selection on their phenotypes, which adapted to different plants, drove speciation (Nosil et al. 2002; Nosil and Crespi 2006; Villoutreix et al. 2023). Comparative genomic analysis of these species revealed significant divergence in approximately 8% of loci, with 1%–2% of loci associated with host adaptation (Nosil et al. 2008). Such phenotypic divergence based on adaptation to different plants as food or oviposition substrates (Drès and Mallet 2002; Matsubayashi et al. 2010) and allochrony in reproductive timing (Taylor and Friesen 2017) are known as major factors in insect speciation. Models with clear ecological isolation have revealed complex evolutionary processes involving the divergence of various phenotypes and genes during niche adaptation. However, these systems are characterized by pronounced ecological differentiation, making it difficult to isolate the earliest genomic signatures of speciation itself. To identify the factors essential for genomic divergence during the early stages of speciation, systems in which clear niche differentiation is absent are indispensable.

The Hawaiian cricket *Laupala* represents a case study of speciation in which geographic and ecological isolation are unclear. They exhibit rapid speciation with gene flow within oceanic islands and display high morphological cryptic variability (Parsons and Shaw 2001; Shaw 2002). Among *Laupala* species, differences in male songs produced by rubbing their forewings together to attract females represent a phenotypic axis of speciation mediated by female sexual selection (Shaw et al. 2007; Ellison et al. 2011; Blankers et al. 2018). However, this speciation axis is not universal within the superfamily Grylloidea, because some species exhibit little song variation, and others lack song entirely. An alternative cricket system without clear ecological and behavioral divergence may reduce the influence of known disruptive selection and highlight the unnoticed evolutionary processes of initial speciation.

The common cricket species *Loxoblemmus campestris* and *L. sylvestris* have widely overlapped distribution in the Japanese archipelago and surrounding regions. The genus *Loxoblemmus* is characterized by diversity in adult male head shape, which is used in male–male fights (Kim et al. 2011; Yoneda et al. 2025). However, these two species exhibit little measurable differentiation in morphology, diet, oviposition substrates, or breeding season despite their overlapping distributions. Here, we test whether these morphologically similar, geographically overlapping taxa represent phylogenetically distinct lineages and investigate how genetic divergence occurs across the entire nuclear genome.

## 2 Materials and Methods

### 2.1 Sample Collection and DNA Sequencing

We collected adult *Loxoblemmus* cricket samples from 19 locations across Japan (Figure 1, Table S1). For DNA sequencing, we extracted genomic DNA from 19 adult female *Loxoblemmus* individuals and one adult male *L. doenitzi*. Hind leg tissues were homogenized, and genomic DNA was extracted using the NucleoSpin DNA Insect kit (TaKaRa). The DNA libraries were sequenced as 150 bp paired‐end reads on the DNBSEQ platform (BGI, Shenzhen, China) (Table S2).

**FIGURE 1:**
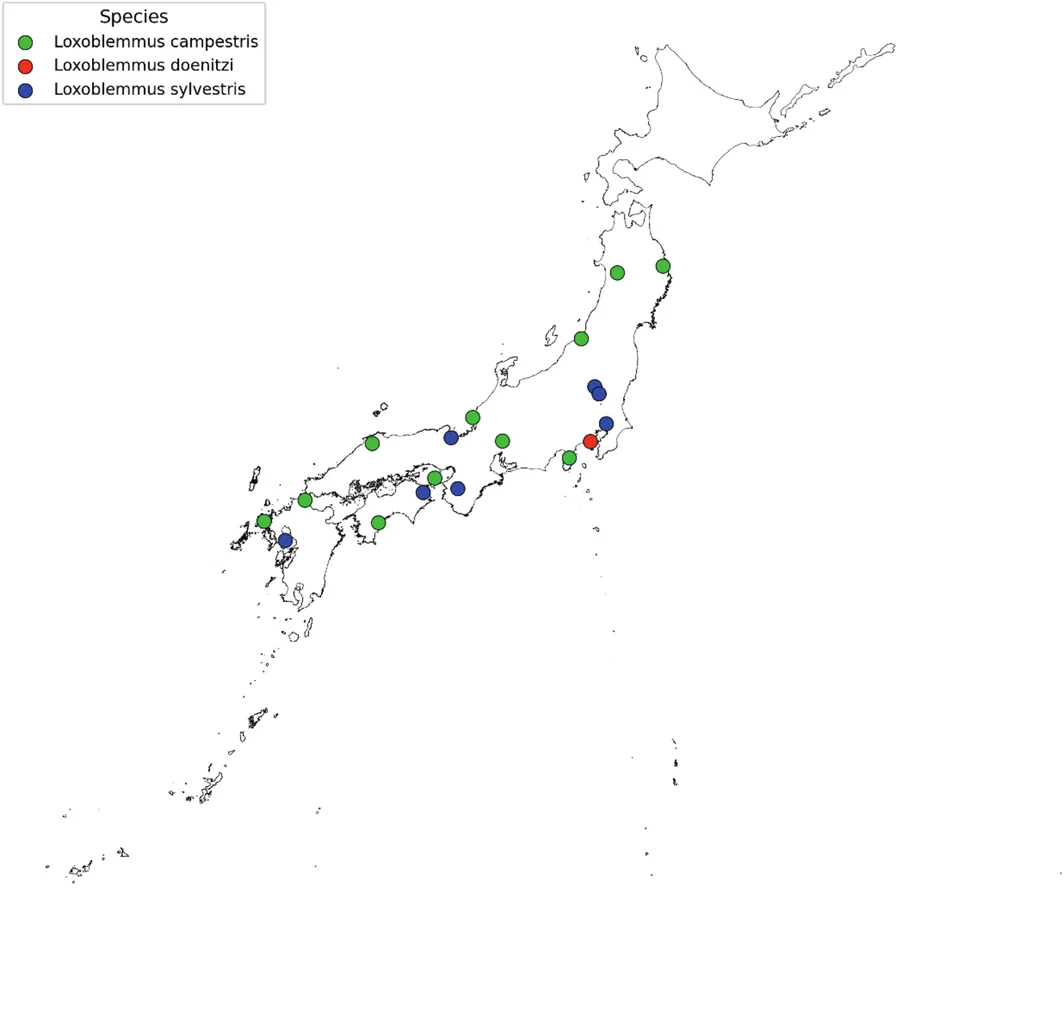
Geographic distribution of sampling locations for *Loxoblemmus campestris*, *L. sylvestris* , and *L. doenitzi* used in this study. A single sampling location was shared between *L. sylvestris* and *L. doenitzi*. Detailed sampling information is provided in Table S1.

### 2.2 Mitochondria Genome Assembly and Gene Annotation

To obtain complete mitochondrial genomes, we assembled the adapter‐trimmed raw reads of all 20 samples by NOVOPlasty v.4.3 (Dierckxsens et al. 2017) or GetOrganelle v.1.7 (Jin et al. 2020) with the default parameters. The obtained complete mitochondrial genome sequences were aligned using MAFFT v.7 (Kuraku et al. 2013). We annotated all 13 protein‐coding genes, 2 rRNAs, and 22 tRNAs using MITOS2 (Donath et al. 2019) on the Galaxy platform (Giardine et al. 2005). The annotation results were visually verified on the multiple alignment of the 20 *Loxoblemmus* samples and mitochondrial genomes of *Gryllus bimaculatus* (MZ440656.1) and *L. doenitzi* (KX057721.1). The circular mitochondrial genomes were drawn using OGDRAW (Greiner et al. 2019).

### 2.3 Species Identification Based on Mitochondrial DNA Sequences

For reliable species identification, additional partial mitochondrial sequences of 16S rRNA ( *L. campestris* LC801728.1 and *L. sylvestris* LC801743.1) and partial cytochrome c oxidase I (*COI*) sequences of *L. arietulus* (LA01, LA02, LA03, LA04, LA05, LA07, LA09, and LA12) were obtained from the NCBI Nucleotide database. The mitochondrial sequences were aligned using Clustal W (Thompson et al. 1994). The neighbor‐joining phylogenetic trees (Saitou and Nei 1987) were inferred using Kimura two‐parameter model (Kimura 1980) and bootstrap analyses were performed on 1000 replicates (Felsenstein 1985) with gap complete deletion in MEGA12 (Kumar et al. 2024).

### 2.4 Mitochondrial Phylogenetic and Phylogeographic Analyses

For molecular phylogenetic analysis, additional mitochondrial genome sequences of Gryllidea cricket species were obtained from NCBI RefSeq database (Goldfarb et al. 2025). The sequences containing compositional anomalies were excluded based on the composition chi‐squared test of IQ‐TREE2 v.2.4.0 (Minh et al. 2020). The species used are summarized in Table S2.

The amino acid sequences of 13 protein‐coding genes were aligned using MAFFT v.7 with “L‐INS‐i” option. The nucleotide sequence multiple alignment was obtained by generating a codon alignment using PAL2NAL v.14.1 (Suyama et al. 2006). The unreliable regions within those alignments were removed using trimAl with “‐gt 0.9” and “‐cons 60” option (Capella‐Gutiérrez et al. 2009). To estimate a molecular phylogenetic tree concatenating 13 protein‐coding genes, PartitionFinder 2.1.1 (Lanfear et al. 2016) was used to select the optimal scheme and estimate the evolutionary model for each partition for both the amino acid sequences and the nucleotide sequences partitioned by the first and second codon positions. Model search was set to “allx,” branchlengths was set to “linked,” and the greedy algorithm was used. Based on the obtained model, we estimated the maximum likelihood phylogenetic tree for the species using IQ‐TREE2 with the “‐b 100,” “‐alrt 1000,” and “‐nt AUTO” options. Furthermore, based on the obtained model, MrBayes v. 3.2 was used to estimate the species Bayesian phylogenetic tree with the options “mcmc ngen = 200000; samplefreq = 1000; sump burnin = 250; sumt burnin = 250” (Ronquist et al. 2012). These phylogenetic trees were visualized using iTOL (Letunic and Bork 2024). To assess the robustness of branches with low support, we additionally inferred amino acid phylogenies using a different dataset that included 33 additional Gryllidea species from GenBank database (Sayers et al. 2025) using the same method (Table S3).

Isolation by distance (IBD) for *L. campestris* and *L. sylvestris* strains was tested using the Mantel test (Mantel 1967) and Pearson correlation analysis with the vegan package (Dixon 2003). The *d*_XY_ values for each sample pair were calculated using MEGA12, and geographic distance used the shortest‐path distance.

### 2.5 Morphological Data Collection and Analysis

The following morphological traits were measured in adult crickets on ice by a single measurer: whole‐body length, forewing length, femur length, and head width. The measurements were taken from 80 male and 178 female *L. campestris* , 16 male and 32 female *L. sylvestris* , and 20 male and 64 female *L. doenitzi* individuals. Data showing discrepancies of 1 mm or more in repeated measurements were excluded, as were data from damaged or defective parts. Differences in these measurements among species and between sexes were tested using the Mann–Whitney *U* test.

### 2.6 Mapping Genomic Read Pairs and Single‐Nucleotide Variant Calling

The paired‐end sequences of 20 samples were filtered using Fastp v.0.23 (Chen et al. 2018). Since there is no reference genome available for the genus *Loxoblemmus*, we used all annotated exon sequences of the *G. bimaculatus* (GCA_017312745.1) as a reference (Ylla et al. 2021). Mapping was performed using BWA‐MEM2 (Vasimuddin et al. 2019) with the default parameters. The SAM files were sorted using the Picard toolkit and converted to BAM files. Mapping coverage for each exon was evaluated using SAMtools' “depth” (Li et al. 2009). After removing duplicate reads with the Picard toolkit's MarkDuplicates, the GATK4 (McKenna et al. 2010) “HaplotypeCaller” was run with the “‐ERC GVCF” option. SNPs were extracted from the called variants using GATK4 and the hard filtering criteria were set as follows: QualByDepth < 2.0, StrandOddRatio > 3.0, FisherStrand > 60.0, RMSMappingQuality < 40.0, MappingQualityRankSumTest < −12.5, and ReadPosRankSumTest < −8.0.

### 2.7 Gene Selection and Quality Filtering

For population and comparative genomic analysis, genome data with gene annotations for 15 Polyneoptera species were obtained from the RefSeq database (Table S4). We predicted single‐copy genes and inferred gene orthology based on sequence homology and phylogenetic trees by running OrthoFinder v.2.5 (Emms and Kelly 2019). Those gene candidates were defined as genes present as single copies in the reference species and in at least 12 species within Polyneoptera samples. Furthermore, we required that the mean and median mapping coverage both be within ±5 (Table S5). Furthermore, using the mapping depth d for all single‐copy gene candidates and the mapping depth $d_{i}$ for each gene, we required that the following three equations be satisfied.

$$\tilde{d}-5<\tilde{d_{i}}\leq \tilde{d}+5$$

$$\bar{d}-5<\bar{d_{i}}\leq \bar{d}+5$$

$$\left|\tilde{d_{i}}-\bar{d_{i}}\right|<5$$

Filtered gene functions and their pathways were predicted using the KEGG database (Kanehisa et al. 2025). KEGG IDs were assigned using the KEGG Automatic Annotation Server (KAAS), with BLAST searches referencing the genomes of *Drosophila melanogaster* , *Apis mellifera* , *Tribolium castaneum* , *Bombyx mori* , *Zootermopsis nevadensis* , and *Caenorhabditis elegans* (Moriya et al. 2007).

### 2.8 Population Genomic Analyses

To evaluate the influence of evolutionary forces on population history and natural selection on the speciation of *L. campestris* and *L. sylvestris* , we performed population genomic analyses on filtered single‐copy nuclear genes. We inferred the phylogenetic relationships of the *Loxoblemmus* strains using Neighbor‐Net with the Jukes‐Cantor model (Jukes and Cantor 1969) in SplitsTree v.6 (Bryant and Moulton 2004). The multispecies coalescent approach implemented by ASTRAL v5.7.1 (Rabiee et al. 2019) was used to consider incomplete lineage sorting (ILS) as a source of phylogenetic heterogeneity across genomic loci. IBD for *L. campestris* and *L. sylvestris* strains was tested using the Mantel test and Pearson correlation analysis with the vegan package. The *d*_XY_ values for each sample pair were calculated using MEGA12, and geographic distance used the shortest‐path distance.

The population structure of the *Loxoblemmus* strains was examined by performing principal component analysis (PCA) using plink v1.9 (Chang et al. 2015) and STRUCTURE/ADMIXTURE analysis using ADMIXTURE v1.3 (Alexander et al. 2009). Variant sites were first filtered based on sequencing depth using BCFtools (Li et al. 2009), retaining genotypes with a read depth of ≥ 5. The filtered VCF file was converted to PLINK binary format, and linkage disequilibrium (LD) pruning was performed using PLINK with the ‐‐indep‐pairwise 50 10 0.1 option.

Genetic differentiation between *L. campestris* and *L. sylvestris* populations was estimated by weighted *F*_*ST*_ value (Hudson et al. 1992) using scikit‐allel v.1.3 in Python. Significance was assessed using an exact population‐label permutation test in which the labels of 18 individuals were exhaustively reassigned while preserving the original group sizes, yielding 31,824 possible assignments. For *F*_*ST*_ values of each gene, one‐sided permutation *p*‐values were calculated and adjusted for multiple testing using Bonferroni correction based on *p*‐value = 0.01 (Bonferroni 1936) and Benjamini–Hochberg (BH) false discovery rate (FDR) procedure (Benjamini and Hochberg 1995).

To investigate lineage‐specific evolutionary rates, we employed the branch model in codeml of PAML v.4.10.1 based on the inferred species trees (Yang 2007). We compared a one‐ratio model (M0), which assumes a single synonymous and nonsynonymous substitution rate ratio for the *L. campestris* and *L. sylvestris* branches with complete deletion of gaps, to a two‐ratio model. Genetic diversity was estimated by *θ*_*w*_ (Watterson 1975), *θ*_*π*_ (Nei and Tajima 1981), and Tajima's *D* (Tajima 1989a, 1989b) using MEGA12. In addition, we investigated patterns consistent with natural selection at the nucleotide level in the fixation of substitutions on genes during the divergence of *L. campestris* and *L. sylvestris* using the McDonald–Kreitman (MK) test and evaluated the results with Fisher's exact test (McDonald and Kreitman 1991) by using PopGenome v.2.7.7 (Pfeifer et al. 2014) and adjusted for multiple testing using BH FDR procedure.

Genes were ranked according to their *F*_*ST*_ values, and KEGG enrichment was evaluated using the gene set enrichment analysis (GSEA) method in clusterProfiler v4.16.0 (Wu et al. 2021). Within this function, adjusted *p*‐values associated with KEGG terms were calculated using the BH adjustment method for multiple comparisons. The adjusted *p*‐values were also used to perform a dot plot analysis using enrichplot v1.28.4 (Xu et al. 2024).

### 2.9 Comparative Genomic Analyses Across Polyneoptera

For comparative genomic analysis, we used 15 Polyneoptera species from the RefSeq database and 20 *Loxoblemmus* strains that we sequenced. The amino acid sequences of the orthologs identified by OrthoFinder were multiple‐aligned using MAFFT with the “L‐INS‐i” option. Those alignments were evaluated based on the completeness score for the alignment (C_a_) from AliStat v.1.15 (Wong et al. 2020). We examined substitutions in each gene based on these amino acid alignments.

We estimated the supermatrix tree for the single‐copy nuclear orthologs across Polyneoptera species, *L. campestris* (LC01), *L. sylvestris* (LS01), and *L. doenitzi* (LD01) using IQ‐TREE2 with the “‐m MFP,” “‐bb 1000,” and “‐nt AUTO” options. The phylogenetic tree was visualized using iTOL. We estimated ancestral amino acids using the IQ‐TREE2 ‐‐ancestral option with “‐asr‐min 0.5,” based on the inferred species tree and the LG + G + F model (Le and Gascuel 2008). Based on the Polyneoptera phylogenetic tree, we calculated the RelTime for Orthoptera species using MEGA12 (Tamura et al. 2018). Based on these distances, we estimated the evolutionary probabilities (*EP*s) for each nuclear amino acid site on each lineage using the LG+G+F model in MEGA12, with *Schistocerca* as the outgroup (Liu et al. 2016). The probability that a low *EP* site exists among the variable amino acid sites for each gene was calculated and adjusted using the Bonferroni correction. For sites with *EP* < 0.05, the BAM files following realignment with GATK were visually inspected using IGV v2.19.8 (Robinson et al. 2011), with particular attention to read depth, consistency of the alternate allele, mapping quality, strand representation, and local alignment context. KEGG enrichment analysis for *EP* values using the over‐representation analysis (ORA) method in clusterProfiler v4.16.0. The background universe consisted of all genes passing the comparative genomic filtering and having an assigned KEGG orthology. The adjusted *p*‐values were also used to perform a dot plot analysis using enrichplot v1.28.4.

## 3 Results

### 3.1 Mitochondrial Phylogenetic Analyses

#### 3.1.1 Mitochondrial Genome Assembly and Gene Annotation

We performed paired‐end short‐read sequencing using DNA extracted from the hind legs of 18 adult individuals of *Loxoblemmus* ( *L. campestris* and *L. sylvestris* ), as well as one male and one female of adult *L. doenitzi* collected from 19 locations in Japan (Figure 1, Tables S1 and S2). Complete circular mitochondrial genomes were successfully assembled de novo for all individuals (Figure 2A–C). All 13 protein‐coding genes, two rRNA genes, and 22 tRNA genes were annotated at homologous positions across samples, and no premature stop codons or frameshift mutations were detected. Although rare polymorphisms in mitochondrial gene order have been reported among Grylloidea species (Sanno et al. 2021), all of the genome sequences determined in this study exhibited the standard Grylloidea gene order.

**FIGURE 2:**
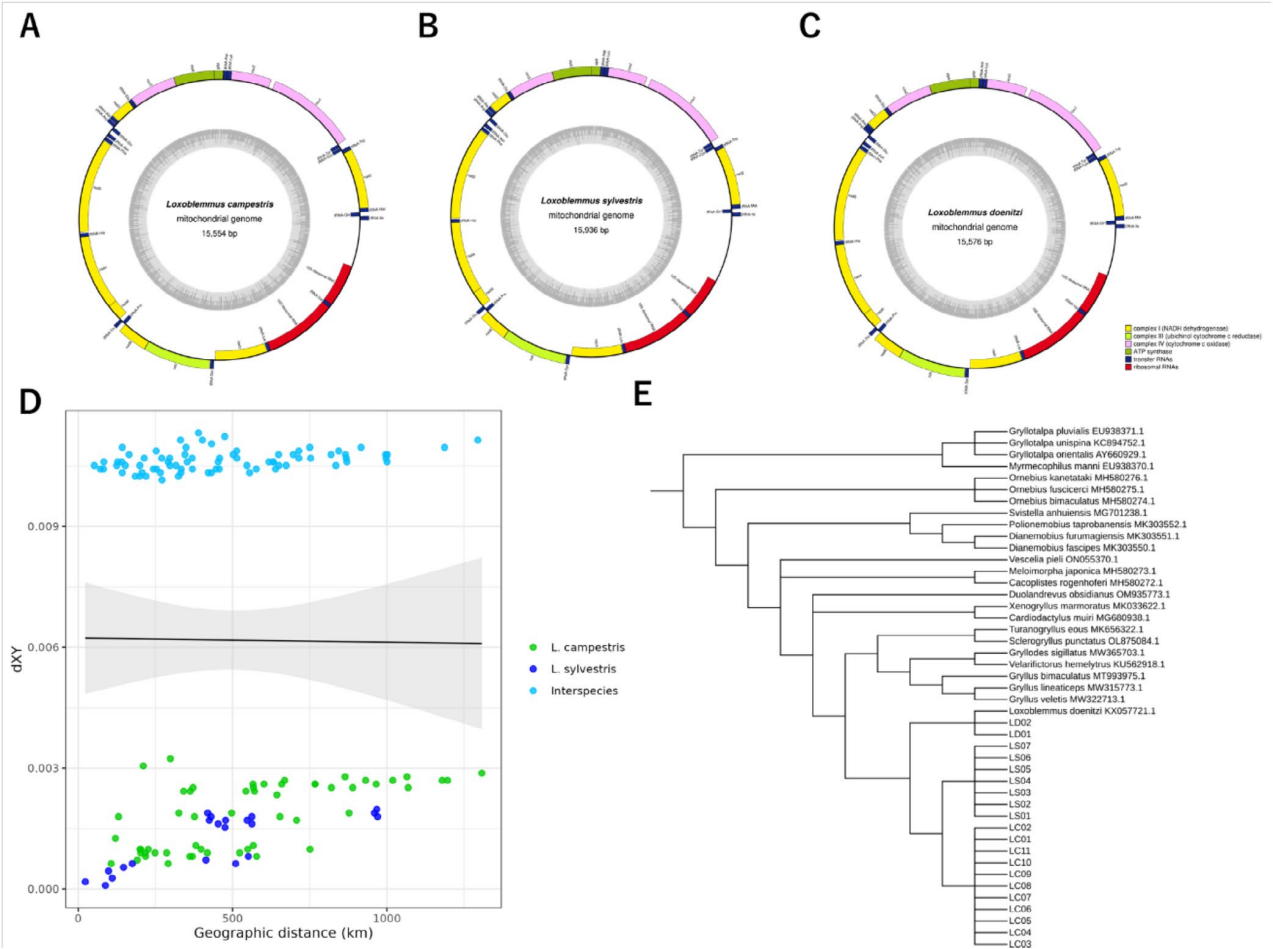
The circular mitochondrial genomes of (A) *L. campestris* (LC01), (B) *L. sylvestris* (LS01), and (C) *L. doenitzi* (LD01). The positions of all annotated protein‐coding genes, tRNAs, and rRNAs are illustrated. (D) Scatter plot of pairwise population genetic distances (*d*_XY_) and pairwise geographic distances among strains of *L. campestris* and *L. sylvestris* . The regression line and the 95% confidence interval (highlighted in gray) are based on the assumption that the two species have not diverged. (E) Mitochondrial phylogeny inferred from concatenated amino acid sequences of all 13 protein‐coding genes. Detailed phylogenetic trees inferred using different methods are shown in Figures S3–S6.

#### 3.1.2 Species Identification of *L. campestris* and *L. sylvestris* Based on Mitochondrial Sequences

We examined whether *L. campestris* and *L. sylvestris* , which have poor morphological differentiation, could be distinguished based on mitochondrial sequence differences. Currently, one partial 16S rRNA sequence is publicly available for each species. Using our sequences and the published sequences of the three *Loxoblemmus* species, we constructed a neighbor‐joining phylogenetic tree aligned to the lengths of the partial sequences (Figure S1). The tree showed that 11 strains clustered with *L. campestris* , 7 strains with *L. sylvestris* , and 2 strains with *L. doenitzi*.

Next, we examined whether *L. campestris* and *L. sylvestris* are geographically isolated populations of the same species. Sampling of *Loxoblemmus* spanned a wide area of the Japanese archipelago and showed no bias (Figure 1). When comparing the correlation between the genetic distance (*d*_XY_) and linear geographic distance for *L. campestris* and *L. sylvestris* , the *d*_XY_ between species was clearly higher than the *d*_XY_ between the respective strains (Figure 2D). When *L. campestris* and *L. sylvestris* were treated as a single population, the correlation coefficient was *r* = −0.006, *R* ^2^ = 0.000042, Mantel *p* = 0.437, and Pearson's *p* = 0.937, indicating no correlation between geographic distance and genetic distance.

There were 112 fixed differences in the mitochondrial genome between *L. campestris* and *L. sylvestris* (Table S6). Among these differences, 8 were in intergenic regions and 39 were at the third position of codons. The 53 fixed mutations at codon Positions 1 and 2, as well as the single fixed in/del at tRNA‐Arg, are genetic markers to distinguish the two species.

The species name *L. campestris* is widely accepted today as a synonym of *L. arietulus*. Using eight publicly available *COI* partial sequences of *L. arietulus*, we also constructed a neighbor‐joining tree (Figure S2). The tree showed *L. arietulus* clustering with *L. doenitzi* rather than with *L. campestris* or *L. sylvestris* . Accordingly, throughout this study, the name *L. campestris* is applied to the taxon identified here as the sister lineage of *L. sylvestris* .

#### 3.1.3 Phylogenetic Relationship of *Loxoblemmus* Within Grylloidea Based on 13 Mitochondrial Genes

The phylogeny of Gryllidea was inferred using concatenated sequences of 13 mitochondrial protein‐coding genes of 20 *Loxoblemmus* strains and of additional species obtained from the RefSeq database. However, the database included sequences whose nucleotide and amino acid compositions were closer to distantly related species than to species within the same genus. Consequently, sequences showing anomalous nucleotide or amino acid composition were excluded based on the composition chi‐squared test (Table S2). As a result, mitochondrial genome sequences of 25 species were added. The phylogenetic tree showed *L. campestris* and *L. sylvestris* as reciprocally monophyletic clades with short branch lengths separating them (Figure 2E). The genus *Loxoblemmus* diverged immediately after the emergence of Gryllinae during the Gryllidea radiation.

On the other hand, phylogenetic relationships other than Gryllinae sometimes altered relationships or showed weaker branch support, depending on whether nucleotide or amino acid sequences were used, or whether maximum likelihood or Bayesian methods were employed (Figures S3–S6). In this study, we concluded that several deep nodes within Gryllinae were weakly supported or contradicted and were therefore represented as a polytomy in Figure 2E. Such instability is consistent with previous reports that orthopteran mitochondrial genomes exhibit diverse GC content even at codon Positions 1 and 2, which can obscure phylogenetic signal in Ensifera (Song et al. 2016; Sanno et al. 2021). However, even in amino acid phylogenetic trees constructed using different sequence sets obtained from the GenBank database, the timing of the genus *Loxoblemmus* divergence within Gryllinae and the independent clusters of *L. campestris* and *L. sylvestris* remained unchanged (Figures S7 and S8).

### 3.2 Morphological Similarity Between *L. campestris* and *L. sylvestris*

Figure 3 shows the appearance of adult males and females of *L. campestris* , *L. sylvestris* , and *L. doenitzi*. The frontal heads of the males of the three species are flat, which is a general characteristic of the genus *Loxoblemmus* (Yoneda et al. 2025). The head shape of *L. doenitzi* is clearly different from the other two species, but the appearances of both males and females of *L. campestris* and *L. sylvestris* are highly similar. *L. sylvestris* has a slightly darker body color and a slightly redder abdomen, but these are not reliable indicators of species identification. The apex of the forewing of *L. campestris* appears slightly flattened, but this characteristic is also difficult to distinguish.

**FIGURE 3:**
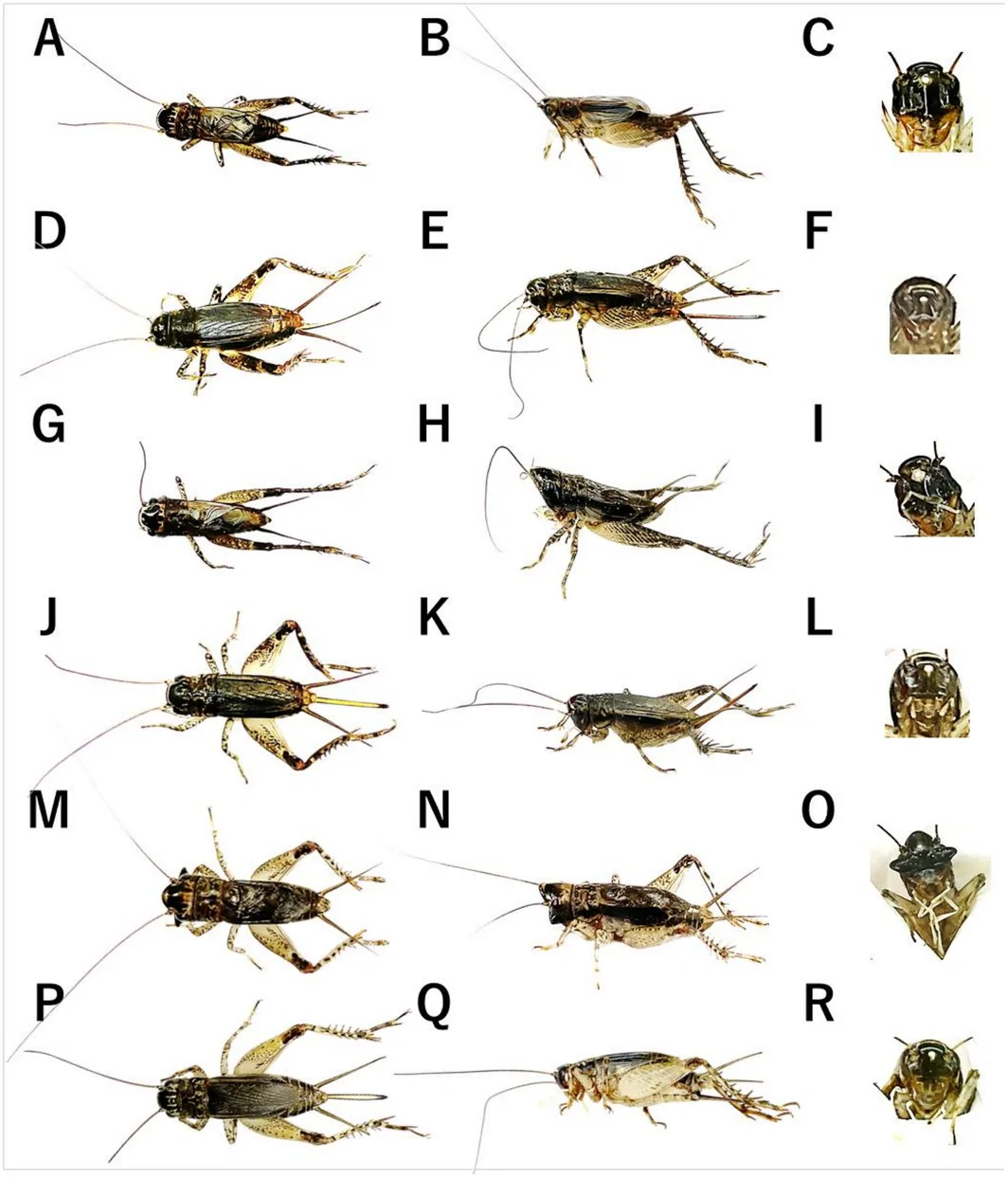
Representative adult morphology is shown for each species and sex. Male *L. campestris* (A–C), female *L. campestris* (D–F), male *L. sylvestris* (G–I), female *L. sylvestris* (J–L), male *L. doenitzi* (M–O), and female *L. doenitzi* (P–R).

To quantitatively evaluate interspecific morphological differences, body length, forewing length, femur length, and head width were measured (Figure 4). As a result, sexual dimorphism was observed in femur length and head width in all species. Forewing length showed sexual dimorphism in *L. doenitzi* but not in *L. campestris* and *L. sylvestris* . Body length and body part sizes were approximately 2 mm larger in *L. doenitzi*, but no clear differences were observed between *L. campestris* and *L. sylvestris* .

**FIGURE 4:**
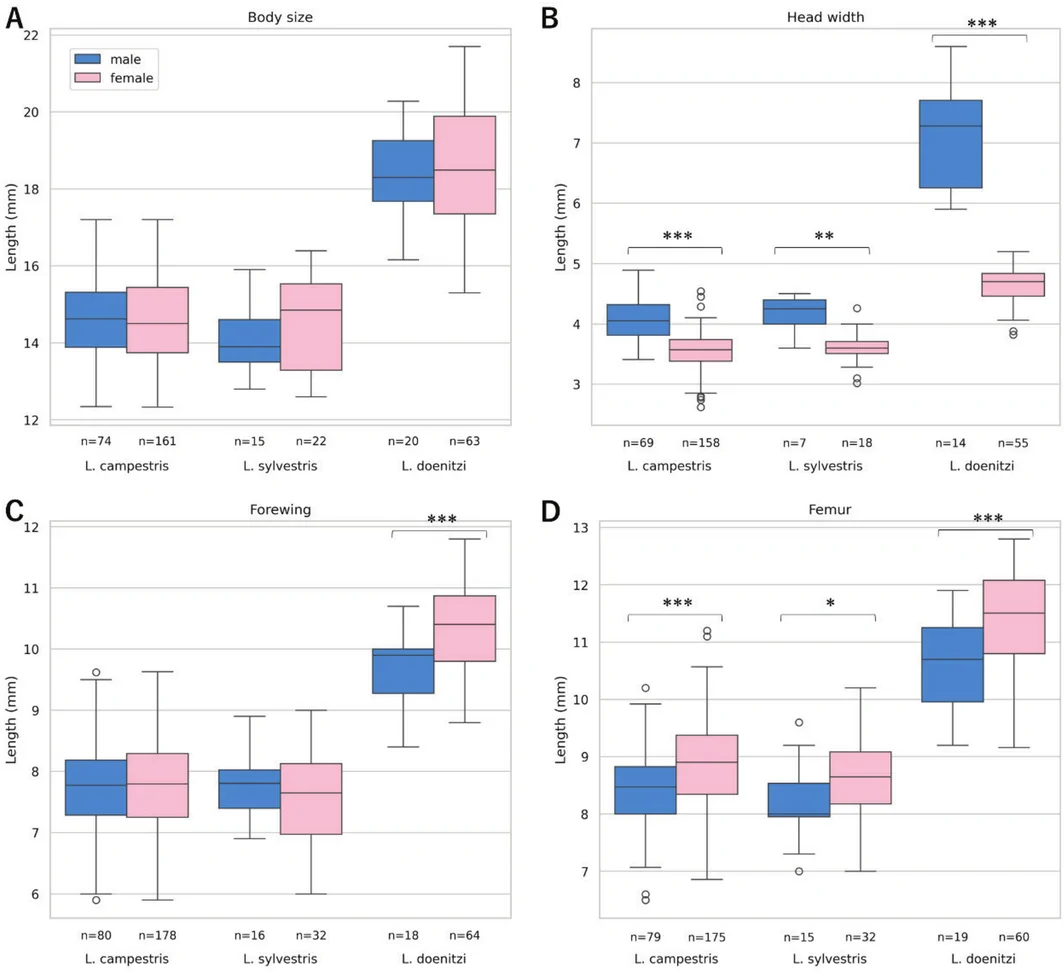
Boxplots showing distributions of body length (A), head width (B), forewing length (C), and femur length (D) of *L. campestris* , *L. sylvestris* , and *L. doenitzi*. Points represent outliers and whiskers indicate the highest and lowest values within 1.5× the interquartile range. Sample sizes are indicated below each box. Sex differences within species were tested using the Mann–Whitney *U* test. *, *p* < 0.05; **, *p* < 0.01; ***, *p* < 0.001.

### 3.3 Nuclear Genomic Analyses

#### 3.3.1 Reference Mapping and Analyzed Gene Set

To investigate the degree of reproductive isolation and genetic divergence between *L. campestris* and *L. sylvestris* , variation analyses were performed using nuclear gene sequences. Paired‐end reads from 20 *Loxoblemmus* strains were mapped to *G. bimaculatus* exonic sequences, and variants were called with hard filtering. Since single‐copy genes in *G. bimaculatus* are not always single‐copy in *Loxoblemmus*, 1376 genes were selected that were single‐copy in at least 12 of the 15 Polyneoptera species obtained from the RefSeq database. The mean and median mapping depths for these genes were 17.6 and 15, respectively. To further reduce the likelihood of retaining duplicated genes and genes with abnormal mapping coverage, ultimately, 1021 genes were carefully selected based on the criteria that their sequence depth fell within ±5 of both the mean and the median, and that the difference between the mean and the median was within 5. These genes constituted the final nuclear gene set used in the subsequent population genomic analyses.

#### 3.3.2 Population Genomic Structure and Genetic Differentiation Between *L. campestris* and *L. sylvestris*

We evaluated relationships among *Loxoblemmus* populations using SNVs on nuclear genes. To investigate phylogenetic relationships considering reticulate evolution, we performed Neighbor‐net analysis. The analysis produced a largely tree‐like pattern, with the three species forming clearly separated clusters (Figure 5A), and no obvious signal of reticulation. Furthermore, the results of both the PCA (Figure S9) and ADMIXTURE/STRUCTURE analysis (Figure S10) indicate clear genetic differentiation between the *Loxoblemmus*, with no obvious evidence of recent admixture. The correlation between genetic distance (*d*_XY_) and linear geographic distance was compared for *L. campestris* and *L. sylvestris* (Figure 5B). When *L. campestris* and *L. sylvestris* were treated as a single population, the correlation coefficient was *r* = −0.049, *R* ^2^ = 0.00238, Mantel *p* = 0.678, and Pearson's *p* = 0.549, indicating no correlation between geographic distance and genetic distance. However, this trend is not as pronounced as it is in mitochondrial genes (Figure 2D). To examine the degree of divergence based on the gene trees, we estimated a multispecies coalescent tree for 20 *Loxoblemmus* strains using ASTRAL, with *G. bimaculatus* as the outgroup (Figure S11). Although *L. campestris* and *L. sylvestris* form distinct clusters, their normalized quartet scores were 47.15 and 45.35, respectively. These results are consistent with genetic differentiation between the two lineages, although they do not rule out low levels of interspecific gene flow or incomplete lineage sorting (ILS). Genome‐wide genetic differentiation between *L. campestris* and *L. sylvestris* was moderate (multilocus *F*_*ST*_ = 0.196). Genetic diversity between *L. campestris* (*θ*_*W*_ = 0.0121, *θ*_*π*_ = 0.0143) and *L. sylvestris* (*θ*_*W*_ = 0.0121, *θ*_*π*_ = 0.0141) and Tajima's *D* between *L. campestris* (Tajima's *D* = 0.750) and *L. sylvestris* (Tajima's *D* = 0.768) were nearly identical.

**FIGURE 5:**
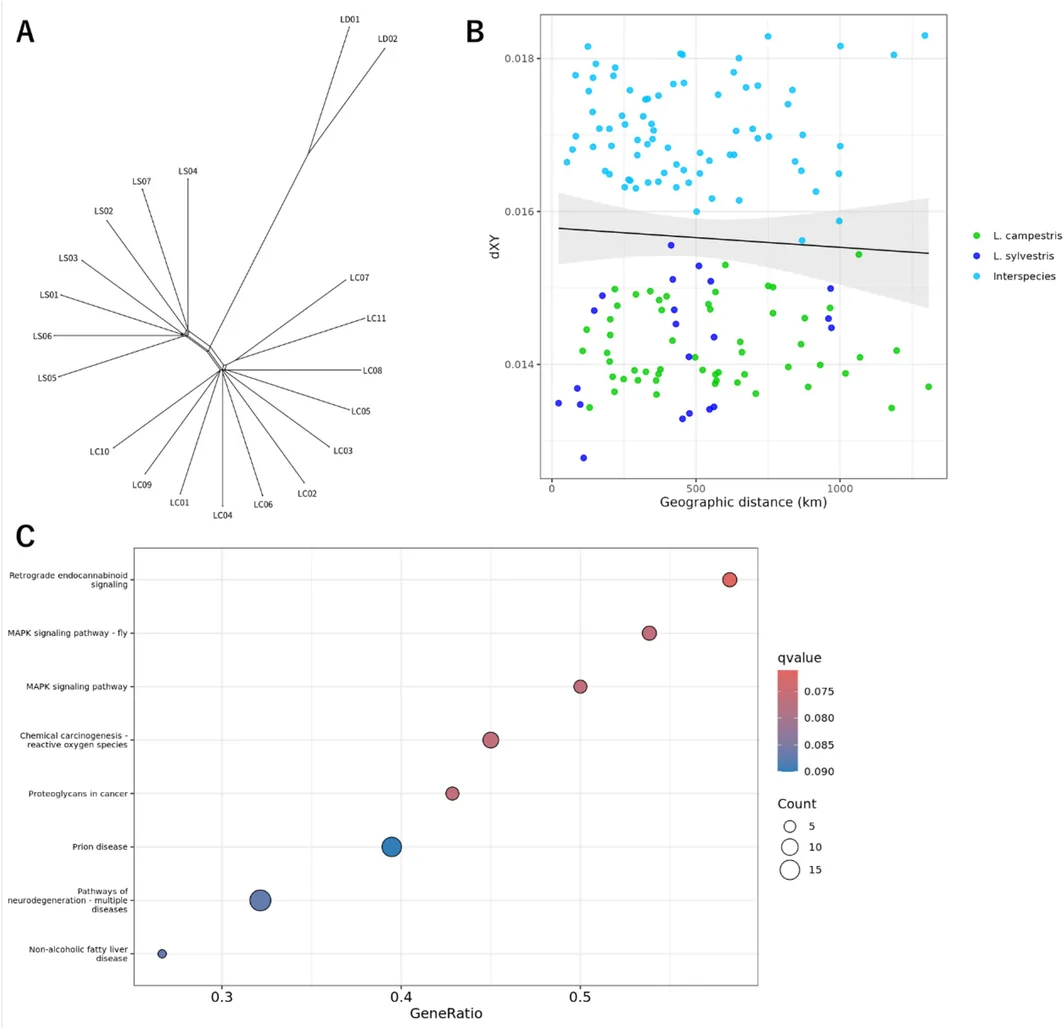
Nuclear genomic analyses of divergence between *L. campestris* and *L. sylvestris* . (A) Neighbor‐net based on Jukes‐Cantor distances calculated from nucleotide sequences of 1021 nuclear genes. (B) Scatter plot of pairwise population genetic distances (*d*_XY_) and pairwise geographic distances among strains of *L. campestris* and *L. sylvestris* . The regression line and the 95% confidence interval (highlighted in gray) are based on the assumption that the two species have not diverged. (C) Dot plot for KEGG gene set enrichment analysis of *F*_*ST*_ between *L. campestris* and *L. sylvestris* .

To identify candidate genes associated with divergence between *L. campestris* and *L. sylvestris* , we evaluated substitution patterns by distinguishing between synonymous and non‐synonymous substitution sites. The average synonymous substitution rate was 0.0139 for *L. campestris* and 0.0117 for *L. sylvestris* , while the average non‐synonymous substitution rate was 0.000940 for *L. campestris* and 0.000858 for *L. sylvestris* (Table S7). The ratio of nonsynonymous to synonymous substitutions was at most 0.482 for the uncharacterized protein (GLH02146.1) in *L. campestris* , and the average was approximately 0.07 for both species. Also, the MK test indicated the signals of positive selection across the entire genome were limited, with only a gene ATP synthase subunit alpha (GLH13744.1) showing a significant deviation from neutrality (*p* < 0.001) (Table S8). Furthermore, for the multiloci *F*_*ST*_ by gene, there were significant differences for 540 genes based on the BH method and 161 genes based on the Bonferroni method; 29% and 53% of these genes, respectively (Table S9), also exhibited low intraspecific genetic diversity (*θ*_*π*_ < 0.01) (Table S10). Using the *F*_*ST*_ values, we performed an exploratory GSEA based on KEGG IDs with *q* < 0.1, which highlighted eight pathways (Figure 5C, Table S11).

#### 3.3.3 Common Amino Acid Substitutions Shared Within *L. campestris* and *L. sylvestris*

To evaluate fixed amino acid substitutions in the context of sequence conservation, we evaluated them through comparative genomic analysis with 15 Polyneoptera species from the RefSeq database. A total of 1021 multiple alignments were filtered based on Alistat's completeness score, resulting in 932 genes. Phylogenetic relationships among the species were inferred using nuclear genes (Figure 6A), which showed the standard topology (Wipfler et al. 2019), thus, the subsequent analyses were performed based on this tree. Among these genes, we detected amino acid substitutions common in the population at 23 sites on 23 genes in *L. campestris* and at 58 sites on 44 genes in *L. sylvestris* (Table S12). These sites were required to satisfy the depth criteria for entire gene.

**FIGURE 6:**
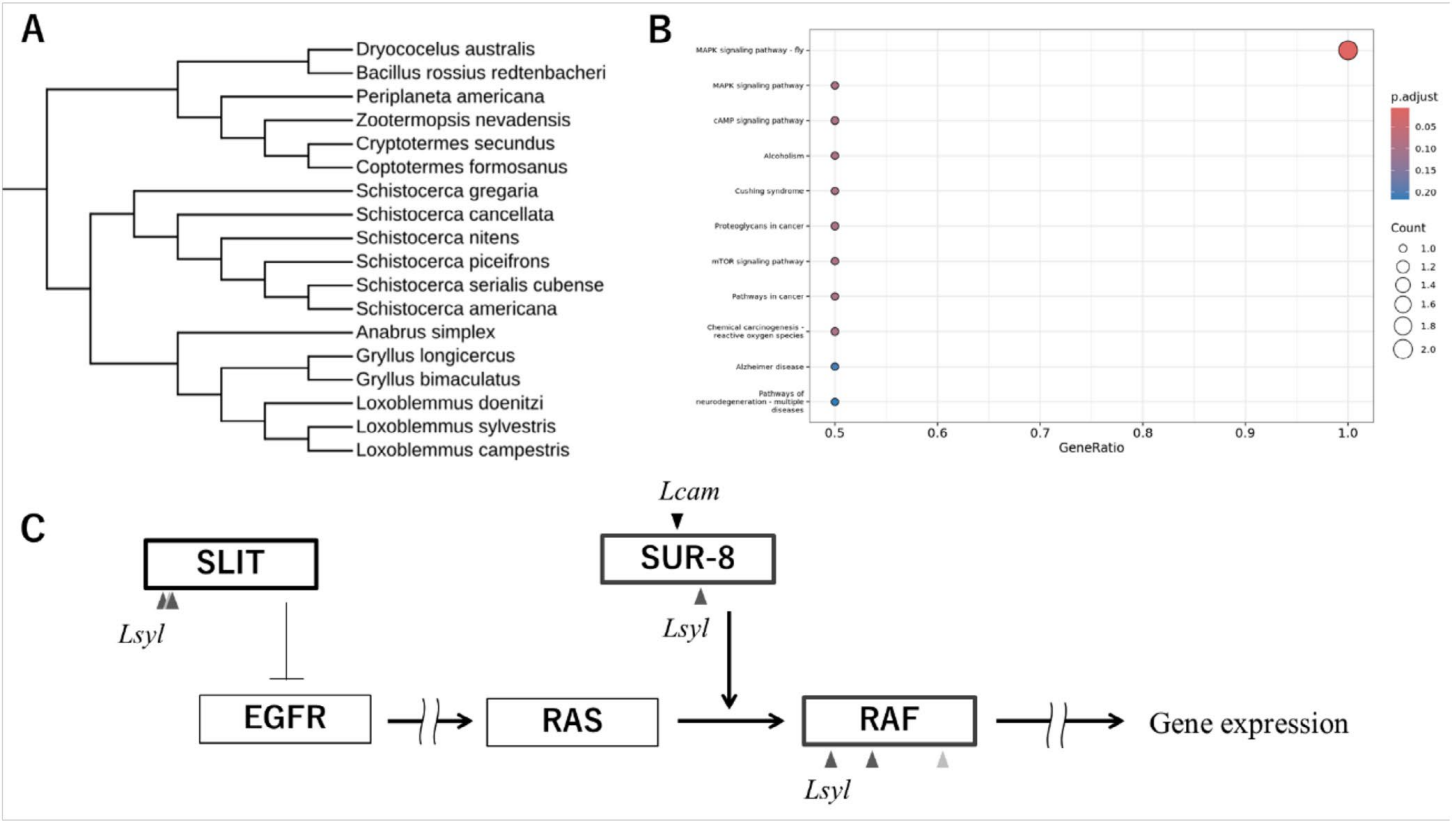
(A) Maximum‐likelihood supermatrix phylogenetic tree inferred from concatenated amino acid sequences of 932 single‐copy nuclear orthologous genes in 15 Polyneoptera species and *Loxoblemmus* species. (B) Dot plot for KEGG over‐representation enrichment analysis of *EPs*. (C) Schematic representations of the Ras/MAPK signaling pathway. Boxed labels indicate proteins. Light gray triangles; *EP* ≥ 0.05, dark gray triangles; *EP* < 0.05, and black triangles; *EP* < 0.01. Lcam, *L. campestris* ; Lsyl, *L. sylvestris* .

To identify substitutions at evolutionarily constrained sites, we evaluated amino acid positions using evolutionary probabilities (*EP*s), where low *EP* values indicate variants that are rarely observed under the inferred neutral model (Liu et al. 2016). We evaluated such amino acid sites in Orthoptera based on *EP*. The results identified 8 sites on 8 genes in *L. campestris* and 13 sites on 11 genes in *L. sylvestris* with *EP* < 0.05 (Table S13). Using the significant *EP* values, we performed an ORA based on KEGG IDs, which highlighted the MAPK pathway (Figure 6B, Table S14). Genes containing multiple such sites were Raf homolog serine/threonine‐protein kinase (*Raf*), leucine‐rich repeat protein soc‐2 homolog (*Sur‐8*), and *Slit*. These substitution sites have also been confirmed through visual inspection of the mappings and interspecies multiple alignments (Figures S12–S29). Note that amino acid residues with low *EP* values in *Slit* appear to be concentrated in the upstream region of this gene due to the incomplete prediction of the *Slit* sequence in the *G. bimaculatus* reference genome. Assuming that the low *EP* sites are randomly distributed across variable amino acid sites, the probability of these genes containing multiple sites is *p* < 0.001 (corrected using the Bonferroni method). All three genes encode components of the Ras/MAPK signaling pathway, including elements of the core signaling complex (Figure 6C). These results indicate that the detected low *EP* amino acid substitutions were non‐randomly concentrated among genes functionally connected within the Ras/MAPK signaling pathway.

## 4 Discussion

This study investigated genetic divergence of two closely related crickets, *L. campestris* and *L. sylvestris* , which have widely overlapping ranges in the temperate regions of the Japanese archipelago and exhibit little morphological divergence. These two crickets show consistent genetic differentiation in both their mitochondrial and nuclear genomes and appear to be closely related cryptic species. Although these two species exhibit moderate levels of genome‐wide genetic differentiation, only a few amino acid substitutions are fixed within each species, indicating that protein‐level divergence remains limited. The small number of genes with evolutionarily specific amino acid substitutions common in one species is not randomly distributed across the genome but instead is enriched in a small set of functionally connected components of the Ras/MAPK signaling pathway. This pattern suggests that early species divergence, lacking clear morphological differentiation, may involve concentrated changes within specific functional modules rather than widespread divergence across the entire genome.

### 4.1 Phylogenetic Relationship and Degree of Genetic Divergence of *L. campestris* and *L. sylvestris*

Both mitochondrial and nuclear phylogenies inferred in this study were consistent with previous studies (Song et al. 2016; Wipfler et al. 2019; Sanno et al. 2021), indicating that the deep phylogenetic relationships within the subfamily Gryllinae remain unresolved and that the genus *Loxoblemmus* diverged early in the Gryllidea radiation. Both mitochondrial and nuclear data indicate that the observed genetic differentiation between *L. campestris* and *L. sylvestris* was not explained by isolation by distance, and the phylogenetic and Neighbor‐Net analyses consistently support their status as distinct evolutionary lineages. However, the alternative quartet supports were approximately 0.5 for both species, and the possibility of ILS and/or low‐frequency interspecific gene flow cannot be ruled out. Although there were inconsistencies in the phylogenetic relationships of mitochondrial and nuclear loci in the parapatric speciation of *Laupala* crickets (Shaw 2002), the geographically overlapping species *L. campestris* and *L. sylvestris* appear to show a clearer phylogenetic separation. However, the first alternative quartet supports were approximately 0.5 for both species; therefore, the possibility of ILS and/or low‐frequency interspecific hybridization cannot be ruled out. Further research is needed to determine whether these lineages diverged under conditions of reproductive isolation or differentiated geographically before subsequent secondary contact. This study found no clear quantitative or qualitative criteria for distinguishing between adult *L. campestris* and *L. sylvestris* . Subtle anatomical morphological or behavioral differentiation, such as in acoustic signals or mating behavior, may be detectable through detailed examination. Although these two species are morphologically very similar, mitochondrial DNA markers can be constructed; thus, clear species identification is possible using DNA sequences. Note that the interpretation that *L. campestris* is a synonym of *L. arietulus* contradicted our molecular phylogenetic analysis; therefore, this study carefully avoids equating *L. arietulus* with *L. campestris* .

*L. equestris* , which is distributed in subtropical regions, is known to be a sister species or subspecies of *L. sylvestris* . While *L. campestris* and *L. sylvestris* may not be the most recent sister species, their level of genetic divergence remains sufficiently low. Using a published orthopteran substitution rate of 14.988 × 10^−9^ substitutions/site/year (Chang et al. 2020) for the mitochondrial 13 protein coding sequence, the time of their divergence was roughly estimated at ~400 thousand years ago. Despite their geographical overlap, moderate genome‐wide differentiation, limited protein‐level divergence at the nuclear level, and few morphological differences, the consistent mitochondrial and nuclear differentiation supports the treatment of *L. campestris* and *L. sylvestris* as distinct evolutionary lineages. Whether complete reproductive isolation has evolved between these lineages remains to be determined. These characteristics make *L. campestris* and *L. sylvestris* a suitable system for studying the early stages of genomic divergence in two cryptic species.

### 4.2 The Genetic Divergence Between *L. campestris* and *L. sylvestris*

In this analysis, we applied filtering criteria based on mapping depth to focus on nuclear genes that were likely to be single‐copy and could be reliably mapped. This may have resulted in an underestimation of the apparent degree of genetic differentiation. While almost all analyzed genes exhibited polymorphism within the species, fixed sites were limited. We identified candidate genes potentially associated with divergence between *L. campestris* and *L. sylvestris* based on two criteria: genes with a high degree of nucleotide sequence divergence by *F*_*ST*_, and genes that have acquired evolutionarily rare amino acid mutations by *EP*s. *EP*s quantify the probability of observing specific amino acids at given sites under a neutral evolutionary model based on phylogenetic conservation. Because this framework relies on cross‐species sequence information rather than within‐population polymorphism, it is applicable to non‐model organisms (Liu et al. 2016). This property allows the identification of candidate adaptive changes even in systems with limited polymorphism.

Although the degree of diversification at the nucleotide level between the two species and the *EP*s for each substituted amino acid site in Orthoptera are measures on different scales, the genes that showed significant differences tended to be concentrated in similar pathways. Retrograde endocannabinoid signaling is significant only when based on the *F*_*ST*_, whereas the MAPK signaling pathway is significant when based on the *EP*s and is also highlighted when based on the *F*_*ST*_. In our dataset, the only genes with multiple amino acid sites of low *EP* are *Raf*, *Slit*, and *Sur‐8*, all of which also have significantly large *F*_*ST*_ distances. These three genes are functionally connected within the Ras/MAPK signaling pathway (Figure 6C), and the concentration of amino acid substitutions in these genes highlights the Ras/MAPK pathway as a candidate functional component of divergence between the two cryptic lineages.

Ras/MAPK signaling pathway regulates various downstream genes and is thought to influence many cellular processes, including development (particularly eye development) (Kumar 2001; Shilo 2014), neural function (Park 2002), and immune responses (Ragab et al. 2011; Tafesh‐Edwards and Eleftherianos 2020). At the center of this pathway, RAF interacts with the RAS1, with SUR‐8 acting as a scaffolding protein (Matsunaga‐Udagawa et al. 2010). Furthermore, SLIT is an inhibitory regulatory gene upstream in this pathway (Kidd et al. 1999). In *Drosophila*, *Slit* is known to be involved in the development of the nervous system and visual system (Kidd et al. 1999; Tayler et al. 2004; Suzuki et al. 2016). Such functional coherence among independently identified genes is notable because these genes are functionally connected within the same signaling pathway. The concentration of candidate substitutions in functionally connected genes is consistent with, but does not demonstrate, coordinated evolution within a functional module. Such patterns suggest that a small number of coordinated molecular changes may contribute to lineage divergence, even when genome‐wide amino acid differentiation is limited and phenotypic differentiation is not readily apparent.

A limitation of this study is the modest sample size per species, which may reduce the precision of population genetic statistics based on within‐species variation. While multilocus estimates were derived from more than 1000 genes and showed consistent patterns, gene‐level estimates should be interpreted with caution. In addition, our nuclear analyses relied on mapping to exonic sequences of *G. bimaculatus* , which may bias against highly divergent regions and result in conservative estimates of genomic divergence. Since our analyses were restricted to filtered orthologs, potential evolutionary processes such as subfunctionalization or neofunctionalization of duplicated genes and differentiations in non‐coding regions are not addressed in the present framework. Furthermore, due to the lack of transcriptomic data, the direct link between genetic changes and phenotypic effects remains inferred. Thus, our results should be interpreted as representing a conservative subset of the genome, in which robust comparative analyses are possible. Within this defined scope, however, we consistently detected non‐random patterns of divergence concentrated in functionally connected genes. Future studies incorporating larger sample sizes, species‐specific reference genomes, and transcriptomic analyses will provide a more comprehensive understanding of genome‐wide divergence and the functional consequences of the identified substitutions.

Our results suggest that the genetic divergence between the cryptic and geographically overlapping lineages of *L. campestris* and *L. sylvestris* is characterized by genome‐wide nucleotide differentiation but relatively few fixed amino acid substitutions. Notably, several amino acid substitutions with low *EP*s occurred in genes functionally connected to the Ras/MAPK signaling pathway, and these genes also showed elevated *F*_*ST*_ values. This convergence of independent signals identifies the Ras/MAPK pathway as a candidate functional component of divergence between the two lineages, although whether these substitutions contributed to reproductive isolation or phenotypic differentiation remains to be determined. In other crickets, *Laupala* species, divergence in male calling and female acoustic preference is a well‐established axis of rapid parapatric speciation, with multiple quantitative trait loci (QTLs) underlying these traits (Shaw et al. 2007; Shaw and Lesnick 2009; Ellison et al. 2011; Xu and Shaw 2021). Whether behavioral traits similarly contribute to divergence in *Loxoblemmus* remains an open question. Another *Loxoblemmus* cricket species, *L. appendicularis*, has three cryptic species in Taiwan (Yeh et al. 2004). Similar genomic analyses of other cryptic *Loxoblemmus* lineages will allow us to evaluate whether the evolutionary patterns of *L. campestris* and *L. sylvestris* represent a shared axis of divergence or one of multiple alternative routes to speciation. Cryptic *Loxoblemmus* species may therefore provide useful systems for understanding the earliest molecular events associated with genomic divergence and speciation.

## Author Contributions

**Kazuhiro Satomura:** conceptualization (lead), data curation (lead), formal analysis (lead), investigation (lead), methodology (lead), project administration (equal), resources (equal), software (lead), supervision (equal), validation (equal), visualization (lead), writing – original draft (lead), writing – review and editing (lead). **Masakazu Saito:** investigation (equal), resources (equal), writing – review and editing (equal). **Atsushi Ogura:** funding acquisition (lead), project administration (equal), supervision (equal), validation (equal), writing – review and editing (equal).

## Funding

This work was supported by Moonshot Research and Development Program, JPJ009237.

## Disclosure

Benefit‐Sharing Statement: All genetic samples were obtained from within Japan in accordance with national law and with respect for local communities and biodiversity.

## Conflicts of Interest

The authors declare no conflicts of interest.

## Supporting information


**Figure S1:** Neighbor‐joining phylogenetic tree of partial 16S rRNA sequences in *Loxoblemmus* species.
**Figure S2:** Neighbor‐joining phylogenetic tree of partial *COI* sequences in *Loxoblemmus* species.
**Figure S3:** Maximum Likelihood phylogenetic tree of amino acid sequences of 13 protein‐coding genes in 25 Gryllidea species obtained from Refseq database and 20 *Loxoblemmus* strains. Numbers above nodes represent bootstrap values (≥ 50)/approximate likelihood ratio test (aLRT) support.
**Figure S4:** Bayesian phylogenetic tree of amino acid sequences of 13 protein‐coding genes in 25 Gryllidea species obtained from Refseq database and 20 *Loxoblemmus* strains. Numbers above branches represent posterior probabilities.
**Figure S5:** Maximum Likelihood phylogenetic tree of nucleotide sequences of 13 protein‐coding genes in 25 Gryllidea species obtained from Refseq database and 20 *Loxoblemmus* strains. Numbers above nodes represent bootstrap values (≥ 50)/approximate likelihood ratio test (aLRT) support.
**Figure S6:** Bayesian phylogenetic tree of nucleotide sequences of 13 protein‐coding genes in 25 Gryllidea species obtained from Refseq database and 20 *Loxoblemmus* strains. Numbers above branches represent posterior probabilities.
**Figure S7:** Maximum Likelihood phylogenetic tree of amino acid sequences of 13 protein‐coding genes in 27 Gryllidea species obtained from GenBank database and 20 *Loxoblemmus* strains. Numbers above nodes represent bootstrap values (≥ 50)/approximate likelihood ratio test (aLRT) support.
**Figure S8:** Bayesian phylogenetic tree of amino acid sequences of 13 protein‐coding genes in 27 Gryllidea species obtained from GenBank database and 20 *Loxoblemmus* strains. Numbers above branches represent posterior probabilities.
**Figure S9:** Principal component analysis (PCA) using variants in 1021 genes. The contribution of PC1 was 7.5%, and that of PC2 was 3.0%.
**Figure S10:** STRUCTURE/ADMIXTURE analysis using variants in 1021 genes, with testing values of K ranging from 3 to 5.
**Figure S11:** Multispecies coalescent tree of 20 *Loxoblemmus* strains. The values were: quartet support; the total number of quartet trees that supporting the main topology; the local posterior probabilities; and the effective number of genes at the common ancestral node (black circle) for *L. campestris* and *L. sylvestris* , respectively. Gbim, *G. bimaculatus* .
**Figure S12:** Reference mapping of site 113 of the *Raf* gene (GLG94720.1) in *L. campestris* (LC01).
**Figure S13:** Reference mapping of site 113 of the *Raf* gene (GLG94720.1) in *L. sylvestris* (LS01).
**Figure S14:** Multiple alignment of amino acid site 113 of the *Raf* gene (GLG94720.1).
**Figure S15:** Reference mapping of site 288 of the *Raf* gene (GLG94720.1) in *L. campestris* (LC01).
**Figure S16:** Reference mapping of site 288 of the *Raf* gene (GLG94720.1) in *L. sylvestris* (LS01).
**Figure S17:** Multiple alignment of amino acid site 288 of the *Raf* gene (GLG94720.1).
**Figure S18:** Reference mapping of site 243 of the *Sur‐8* gene (GLH15814.1) in *L. campestris* (LC01).
**Figure S19:** Reference mapping of site 243 of the *Sur‐8* gene (GLH15814.1) in *L. sylvestris* (LS01).
**Figure S20:** Multiple alignment of amino acid site 243 of the *Sur‐8* gene (GLH15814.1).
**Figure S21:** Reference mapping of site 324 of the *Sur‐8* gene (GLH15814.1) in *L. campestris* (LC01).
**Figure S22:** Reference mapping of site 324 of the *Sur‐8* gene (GLH15814.1) in *L. sylvestris* (LS01).
**Figure S23:** Multiple alignment of amino acid site 324 of the *Sur‐8* gene (GLH15814.1).
**Figure S24:** Reference mapping of site 153 of the *Slit* gene (GLH17197.1) in *L. campestris* (LC01).
**Figure S25:** Reference mapping of site 153 of the *Slit* gene (GLH17197.1) in *L. sylvestris* (LS01).
**Figure S26:** Multiple alignment of amino acid site 153 of the *Slit* gene (GLH17197.1).
**Figure S27:** Reference mapping of site 232 of the *Slit* gene (GLH17197.1) in *L. campestris* (LC01).
**Figure S28:** Reference mapping of site 232 of the *Slit* gene (GLH17197.1) in *L. sylvestris* (LS01).
**Figure S29:** Multiple alignment of amino acid site 232 of the *Slit* gene (GLH17197.1).

**Table S1:** List of *Loxoblemmus* strains used in this study.
**Table S2:** Species list and composition chi‐squared test for cricket species from NCBI RefSeq database and *Loxoblemmus* strains.
**Table S3:** Species list and composition chi‐squared test for cricket species from Genbank database and *Loxoblemmus* strains.
**Table S4:** Sample list of Polyneoptera species from NCBI RefSeq database.
**Table S5:** Mean and median mapping coverage.
**Table S6:** Fixed differences in the mitochondrial genome between *L. campestris* and *L. sylvestris* .
**Table S7:** Ratios of nonsynonymous and synonymous substitutions for 1021 genes of *L. campestris* and *L. sylvestris* .
**Table S8:** McDonald–Kreitman test for 1021 genes of *L. campestris* and *L. sylvestris* .
**Table S9:** ece374457‐sup‐0002‐Tables.xlsx. *F*_*ST*_ values for 1021 genes of *L. campestris* and *L. sylvestris* . *, *p* < 0.05; **, *p* < 0.01.
**Table S10:** The *θ*_*W*_, *θ*_*π*_ and Tajima's *D* values on the 1021 genes of *L. campestris* and *L. sylvestris* .
**Table S11:** Gene set enrichment analysis for *F*_*ST*_ between *L. campestris* and *L. sylvestris* .
**Table S12:** Number of fixed amino acid sites on the 932 genes of *L. campestris* and *L. sylvestris* .
**Table S13:** Evolutionary probabilities (*EP*) for fixed amino acid sites on the 932 genes of *L. campestris* and *L. sylvestris* .
**Table S14:** Over‐representation analysis for evolutionary probabilities (*EP*s) of *L. campestris* and *L. sylvestris* .

## Data Availability

The mitochondrial genomes for this article have been deposited at DDBJ under the accession numbers LC919104‐LC919123 under the BioProject PRJDB40271.
